# High-dose rate brachytherapy in localized penile cancer: short-term clinical outcome analysis

**DOI:** 10.1186/1748-717X-9-142

**Published:** 2014-06-19

**Authors:** Yohann Rouscoff, Alexander Tuan Falk, Matthieu Durand, Jocelyn Gal, Marie-Eve Chand, Mathieu Gautier, Alexandre Marsaud, Daniel Chevallier, Jean Amiel, Jean-Michel Hannoun-Levi

**Affiliations:** 1 Department of Urology, University of Nice Sophia-Antipolis, Hôpital Archet 2, Centre hospitalier universitaire de Nice, Nice, France; 2 Department of Radiation Therapy, Antoine Lacassagne Cancer Center and University of Nice-Sophia, Nice, France; 3 Biostatistic Unit, Antoine Lacassagne Cancer Center, Nice, France

## Abstract

**Purpose:**

To assess clinical outcomes of high-dose rate interstitial brachytherapy (HIB) in localized penile carcinoma.

**Material and methods:**

From 03/2006 to 08/2013, patients with biopsy-proven T1-T2 (<4 cm) non-metastatic localized penile squamous cell carcinoma underwent HIB. Under general anaesthesia, after Foley catheter placement, needles were placed in the target volume using a dedicated template. Planification was carried out with a post-implant CT-scan to deliver a total dose of 36 Gy in 9 fractions over 5 days (in adjuvant setting) or 39 Gy in 9 fractions over 5 days (as monotherapy). Dose-volume adaptation was manually achieved using graphical optimization. Dosimetric data and clinical outcomes were retrospectively analyzed. Toxicities were graded using the CTC v4.0.

**Results:**

With a median follow-up of 27 months [5.1-83], 12 patients including 8 T1a, 3 T1b and 1 T2 N0 underwent HIB (sole therapy: 11 pts; adjuvant: 1 pt). The actuarial 5-year relapse-free, cause-specific and overall survival rates were 83%, 100% and 78% respectively. Comparing pre and post treatment evaluation, no IPSS or IIEF-5 changes were reported. Dermatitis was reported systematically 1 month after HIB including 6 G1, 5 G2 and 1 G3. Only 1 experienced long-term G3 successfully treated with hyperbaric oxygen therapy. One urethral meatus stenosis G3 required meatotomy.

**Conclusion:**

In selected patients with T1-T2 localized penile cancer, HIB may be considered as an optional conservative therapy. Longer follow-up is needed to confirm these encouraging preliminary results.

## Introduction

With a yearly incidence of 1 out of 100 000 men in Europe and the United States, penile cancer remains a rare disease. Historically, the standard treatment for localized tumors is partial amputation bringing psychological distress for the men’s body [1,2]. For this reason, conservative treatment comparable to the management of breast cancer has been developed with the principle of maintaining organ function while maintaining oncological results. These conservative treatments are based on close collaboration between the surgeon and the radiation oncologist both in terms of therapeutic approach and follow-up. Indeed, the organization of an alternate monitoring between the urologist and radiation oncologist allows the patient to benefit from two distinct expertise centered on the same pathology.

Low-dose rate (LDR) brachytherapy is a validated therapeutic modality in localized penile tumors T1-2 < 4 cm [3,4]. For radioprotection and dose distribution optimization considerations as well as therapeutic comfort of the patient, high-dose rate brachytherapy (HDB) has become a standard treatment in many cancers (cervical [5], breast [6] and prostate [7]). The aim of our study was to evaluate the clinical outcome of HDB for penile cancers classified T1a, T1b or T2 < 4 cm according to the TNM 2009 [8].

## Materials and methods

We performed a retrospective, single-center, descriptive study evaluating the results of HDB performed for patients with a localized penile cancer.

### Patient characteristics

From March 2006 to August 2013, at the Centre Antoine Lacassagne (Nice, France) with the collaboration of the Urology department of the Nice University Hospital, 12 patients (pts) with histologically confirmed penile cancers were treated by HDB. Preoperatively, all patients underwent a complete physical examination to assess the depth extension completed if needed by a penile MRI. An ultrasound of the inguinal area (without or with biopsy in suspected cases of lymphadenopathy) and abdominopelvic CT were performed to assess nodal and metastatic status in accordance with the recommendations of the Cancer Committee of the French Association of Urology (AFU) [4].

All patients presented a squamous cell carcinoma of the penis. Circumcision was required prior to brachytherapy and performed for all patients. Excised tissue pathological reports (biopsies or lumpectomy) were used to determine tumor staging. Patient characteristics are reported in Table 1. No patient had lymph node or metastatic disease spreading. Prior to the treatment process, patients were given detailed information about current data, advantages and disadvantages of this conservative technique. This therapeutic approach was initially approved by a local ethics committee.

**Table 1:** Clinical, technical and dosimetric data

**Data**	**Median**	**Min - Max**
Age (year)	77	[47–84]
Tumour size (mm)	25	[9–32]
Pre-op IPSS	2	[0–18]
Pre-op IIEF5	16	[5–25]
Time interval S/B (months)	120	[18–364]
Follow-up (months)	27	[5.1 - 83]
Total delivered dose (Gy)	38.5	[34–43]
EQD2_αβ3_ (Gy)	57.5	[47–68]
EQD2_αβ10_ (Gy)	46	[41–54]
Dose/f (Gy)	4	[3.5 - 4.5]
# of fractions	9	[7–10]
# needles	9	[3–12]
# plans	3	[3]
CTV (cc)	12.1	[4–42]
D90 (%)	106	[83–118]
V100 (%)	93	[78–99]
V150 (%)	40.5	[29–57]
V200 (%)	15	[11–22]
DHI	0.5	[0.46 - 0.67]
D10u (%)	126	[59–217]
D30u (%)	115	[27–177]

Pre-op IPSS: pre-operative *International Prostate Symptom Score*; Pre-op IIEF5: pre-operative *International Index of Erectile Function*; Time interval S/B: Time interval between surgery and brachytherapy; EQD2_αβ3_: equivalent dose at 2 Gy for normal tissues (αβ3); EQD2_αβ10_: equivalent dose at 2 Gy for tumour (αβ10); Dose/f: dose per fraction; # of fractions: total number of fraction; # Needles: total number of needles; # Plans: total number of plans; CTV: clinical target volume; D90: dose delivered to 90% of CTV expressed in percentage of the prescribed dose; V100: CTV receiving 100% of the prescribed dose expressed in percentage; V150: CTV receiving 150% of the prescribed dose expressed in percentage; V200: CTV receiving 200% of the prescribed dose expressed in percentage; DHI: Dose Homogeneity Index = [V100 – V150]/V100; D10u: dose delivered to 10 cc of the urethral volume expressed in percentage of the prescribed dose; D30u: dose delivered to 30 cc of the urethral volume expressed in percentage of the prescribed dose.

### HDR brachytherapy planification

**Figure 1:**
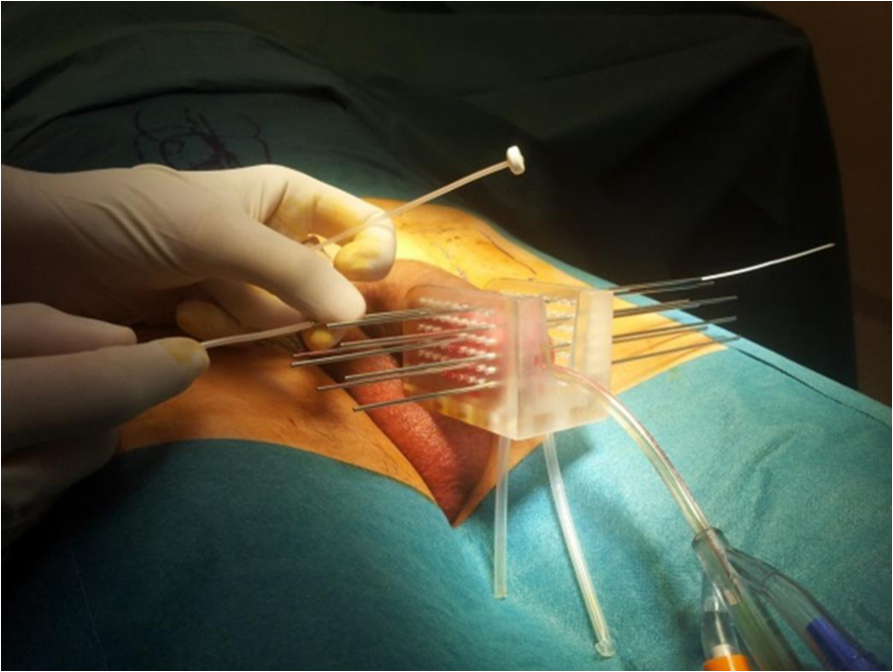
Intra-operative view showing the placement of the needles through the dedicated template.

After urethral catheterization with a CH 18 Foley catheter, the penis was placed in a dedicated applicator with two parallel templates with evenly 10 mm spaced out holes in all directions (Figure 1). The target volume was clinically determined. Needles insertion allowed plastic catheters placement (*Sharp Needles*™; Nucletron, an Elekta company, Elekta AB, Stockholm, Sweden) through the templates in regard to the tumor volume in 1 to 3 plans (depending on the target volume to be treated), with special attention to not damage the urethra.

**Figure 2:**
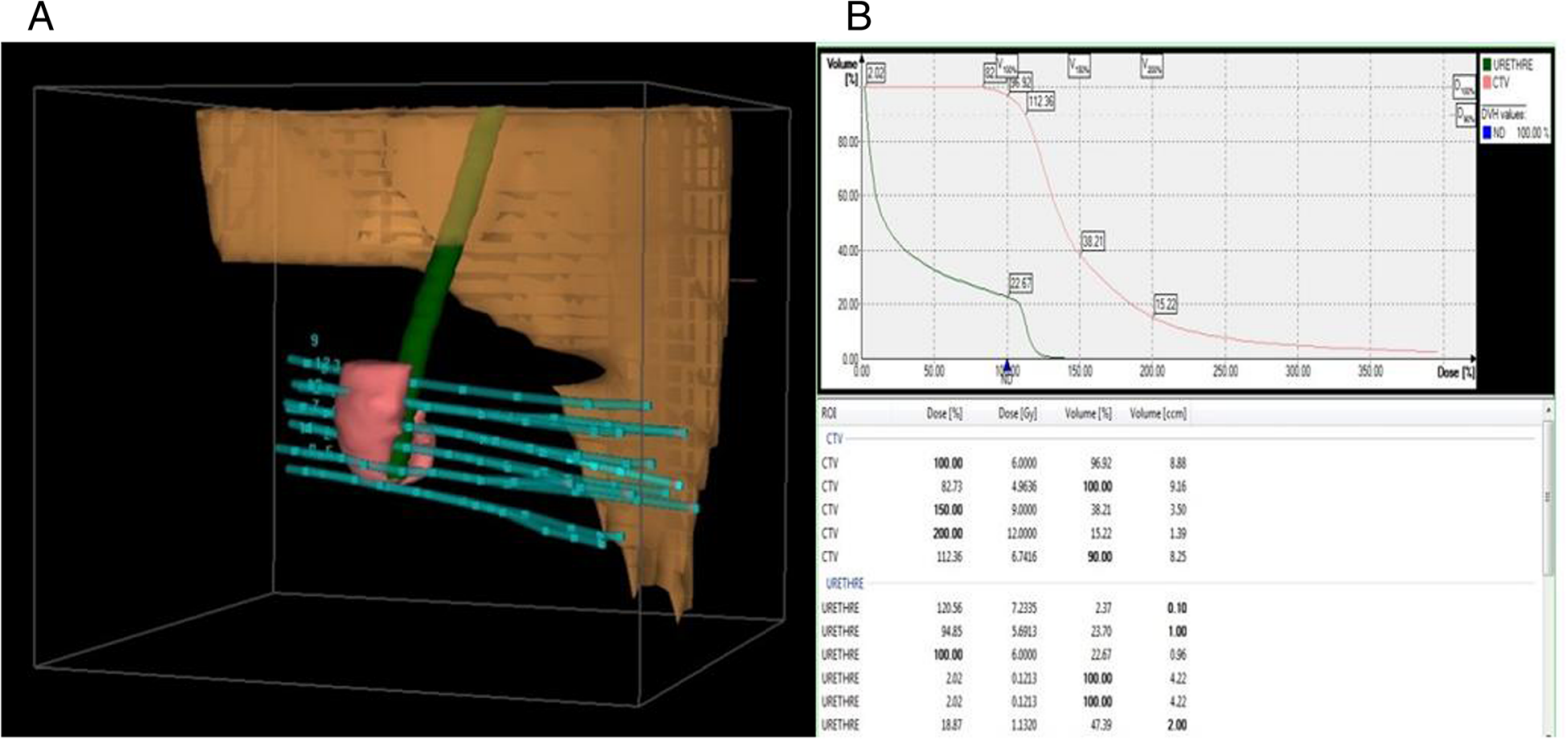
Post-implant CT-scan 3D reconstruction (A), dose-volume histogram (B).

After patient recovery, CT-scan planification (without iodine injection) was performed for the dose distribution analysis and optimization. After delineation of the Clinical Target Volume (CTV) and the organs at risk (mainly the urethra) (Figure 2A), dose-volume adaptation was manually achieved by dwell location and time variation (graphical optimization) (*OncentraBrachy™*; Nucletron an Elekta Company, Stockholm, Sweden) (Figure 2B). Dose constraints used for the CTV included V100% (volume receiving 100% of the prescribed dose) > 95% of the prescribed dose, V150% (volume receiving 150% of the prescribed dose) < 35% of the prescribed dose. Confluence of two V200% isodoses (volume receiving 200% of the prescribed dose) and V200% diameter > 10 mm were avoided. Dose constraints used for the urethra were V115% < 1% (urethra volume receiving 115% of the prescribed dose should be less than 1% of the urethra volume). The prescribed dose depended on the indication: for postoperative brachytherapy, a total dose of 36 Gy in 9 fractions over 5 consecutive days was delivered (6 Gy at day 1 then 2 × 3.75 Gy twice daily from days 2 to 5); for brachytherapy applied as definitive treatment, a total dose of 39 Gy in 9 fractions over 5 consecutive days was delivered (7 Gy at day 1 then 2 × 4 Gy twice daily from days 2 to 5. An interval of at least 6 hours was observed between the 2 daily fractions. This regimen was adapted from a series of preoperative HDR brachytherapy used for cervical cancer for which a total dose of 39 Gy in 9 fractions was delivered over 5 days leading to a complete pathological response rate observed on the post-operative specimen of 92% for squamous cell carcinoma [5].

### HDR brachytherapy delivery

The treatment takes place in a bunker while the patient remains lying in his bed. The length of each fraction depends on the number of implanted vectors and the activity of the radioactive source at the time of the treatment and is around ten minutes. Throughout the duration of hospitalization, the patient remains in a conventional non-shielded room. The treatment is performed in optimal conditions of radioprotection for the medical staff and the patient’s family (visits possible). Indeed, the patient is carrying a radioactive source only during the few minutes of treatment in the morning and the afternoon. These conditions allow making the necessary care (prevention of decubitus complications, local care…) safe for nursing staff.

After the last irradiation session and patient premedication (paracetamol, tramadol, and midazolam), all the needles and the urinary catheter were removed. The patient left the hospital with a prescription for local care for radio dermatitis localized in the irradiated area. It usually occurred in the week following the end of treatment and can last up to 4 to 8 weeks (clear and precise information is given to the patient regarding the occurrence of acute skin side effects).

### Dosimetric analysis

Equivalent dose at 2 Gy for αβ ratio = 10 (EQD2αβ10 for tumor tissue) and for αβ ratio = 3 (EQDαβ3 for healthy tissue) were calculated. D90 (dose delivered to 90% of CTV, expressed as a percentage of the prescribed dose) and V100 (volume receiving 100% of the prescribed dose, expressed as % of CTV), V150 (volume receiving 150% of the prescribed dose expressed as % of CTV) and V200 (volume receiving 200% of the prescribed dose, expressed as % of CTV) were studied. DHI (Dose Homogenity Index) and D10u (dose delivered to 10% of the urethral volume) and D30u (dose delivered to 30% of the urethral volume) were also reported.

### Analysis of oncological results

A clinical examination of the penile and inguinal areas was conducted and completed if necessary with an inguinal ultrasound exam, penis MRI or positron emission tomography (PET) using fluorine 18-labeled fluorodeoxyglucose. Treatment failure (post-treatment relapse) was defined as a biopsy of the treated zone confirming local recurrence of the disease or the occurrence of nodal or metastatic disease. Local recurrence-free survival, overall and specific survival rates were calculated. The median time to onset of relapse was calculated from the date of treatment and the date of onset of recurrence. For disease-free survival, 2 situations were analyzed: local recurrence and/or regional lymph node recurrence (inguinal and/or iliac).

### Analysis of functional results

A reference pre-treatment status for urinary (*International Prostate Symptom Score* - IPSS), and sexual (*International Index of Erectile Function* 5-item - IIEF -5) functions as well as skin status were established. Post-treatment toxicities were scored according to CTCv4.0 classification [9]. At each follow-up visit (1, 6, 12 and 24 months), the evaluation of urinary function was based on the IPSS and the presence or absence of urethral stricture. Sexual function was analyzed with the IIEF-5 score. The skin condition was analyzed according to clinical examination focusing on assessing scarring, pigmentation, telangiectasia, induration or tenderness into the irradiated volume. The rate of penile preservation at the end of follow-up was also calculated.

### Statistical analyzes

Data were analyzed using software R3.0.1. Quantitative data were represented as median, extreme, mean and standard deviation. Qualitative data were represented as frequency, percentage and 95% confidence interval. Overall survival was defined as the time between the date of diagnosis and death from any cause. Local recurrence-free and/or inguinal-free survivals were defined as the time between the date of diagnosis and date of local recurrence or the date of inguinal recurrence. These data were estimated and represented at different time intervals with their 95% confidence using the Kaplan-Meier method. Patients were censored at the time of death or at last follow-up. A matched analysis by a nonparametric Friedman test was used. The level of significance was set at a p-value < 0.05.

## Results

With a median follow up of 27 months [5.1 - 83], the median age was 77 years [47–84]. According to the TNM UICC classification, patients were classified [8]: pT1a (8 pts - 66%), pT1b (3 pts - 25%) and pT2 (1 pt - 9%). The median tumor size was 25 mm [9–32]. Nine patients (75%) had a tumor of the glans, 3 pts (25%) had involvement of the coronal sulcus. The median time between surgery and brachytherapy was 120 days [18–364]. Median pre-operative IPSS and IIEF-5 scores were respectively 2 [0–18] and 16 [5–25].

### Dosimetric results

The median CTV was 12.1 cc [4–42], the median D90 was 106% [83–118]. The median dose per fraction was 4 Gy [3.5 - 4.5] with a median number of fractions of 9 [7-10] (Table 1). The median total delivered dose was 38.5 Gy [34–43] corresponding to a EQD2αβ10 of 46 Gy [41–54] and EQDαβ3 of 57.5 Gy [47–68]. The median V100, V150 and V200 were 93% [78–99], 40.5% [29–57] and 15% [11–22] of the CTV. The median DHI was 0.5 [0.46 - 0.67]. The median D10u and D30u were respectively 126% [59–217] and 115% [27–177].

### Oncological results

**Figure 3:**
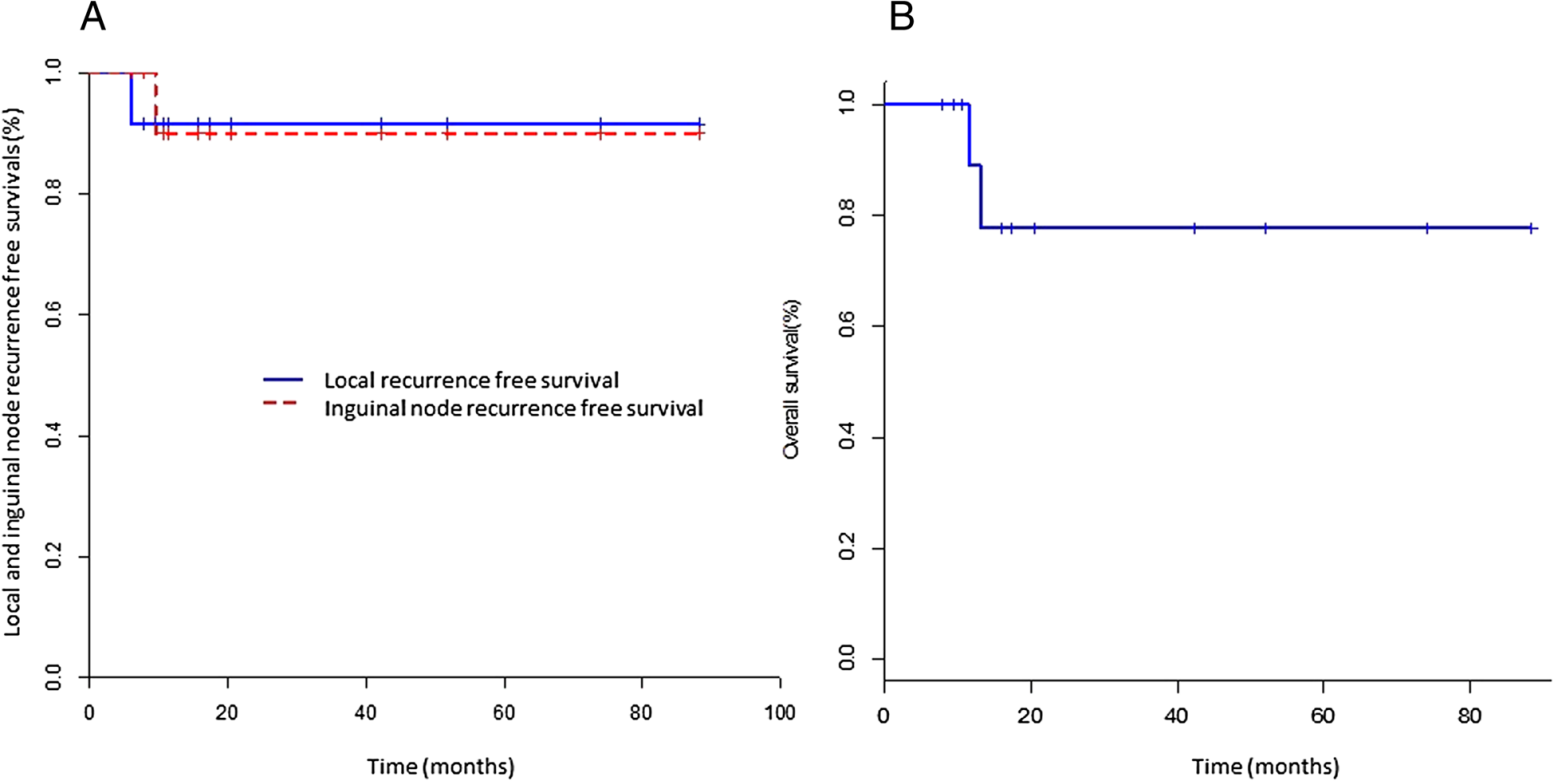
Kaplan-Meier curves for local and inguinal recurrence-free survival (A) and overall survival (B).

Two patients (17%) presented an early loco-regional recurrence at 12 months. The first one developed an histologically confirmed right inguinal lymph node without local recurrence, (treated by salvage external beam radiotherapy). The second one presented an isolated local recurrence in the irradiated zone treated by salvage total amputation of the penis. The mean time to loco-regional recurrence was 7.7 months [6–9.4]. Because all the events occurred during the first 18 months, the actuarial rate of loco-regional recurrence free survival, specific and overall survival at 2, 3 and 5 years are the same. The 5-year actuarial loco-regional recurrence free survival rate was 83% (95% [63–100]) (Figure 3a), while the 5-year actuarial overall survival rate was 78% (95% [55–100]) (Figure 3b). The 5-year actuarial specific actuarial rate was 100%, with 2 deaths being due to intercurrent causes.

### Functional results

**Figure 4:**
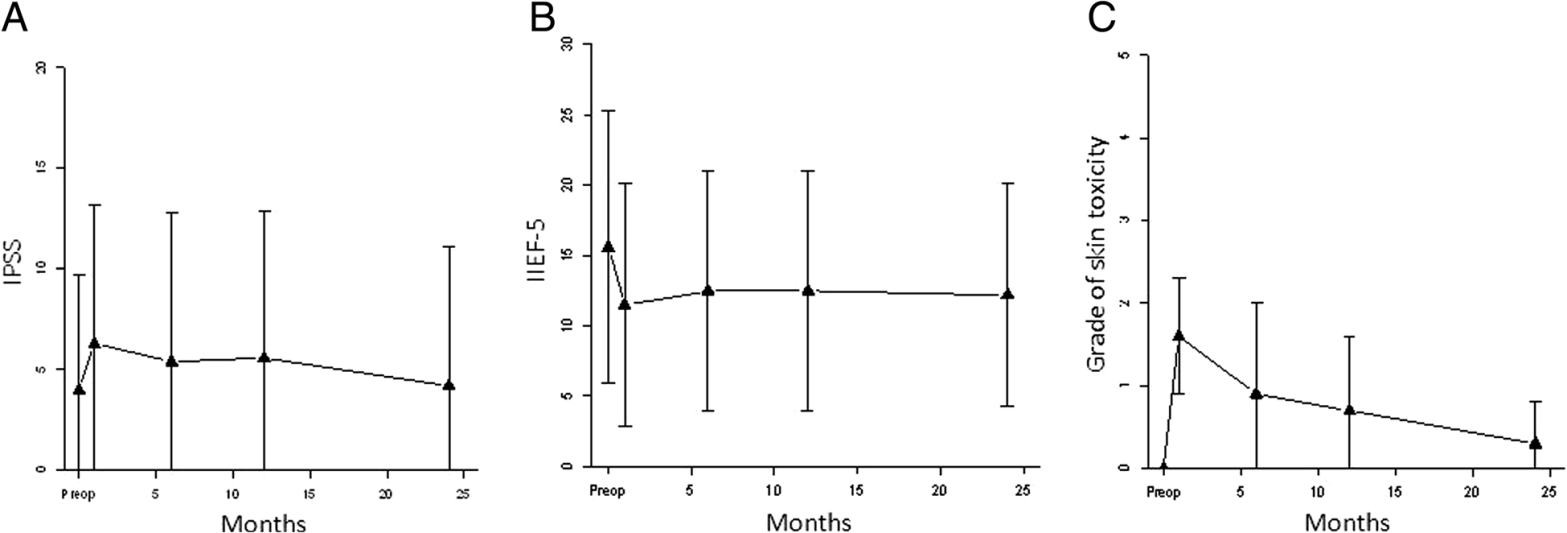
Evaluation of urinary (A) and sexual (B) functional outcomes after interstitial high-dose rate brachytherapy and skin toxicity evaluation (C).

**Figure 5:**
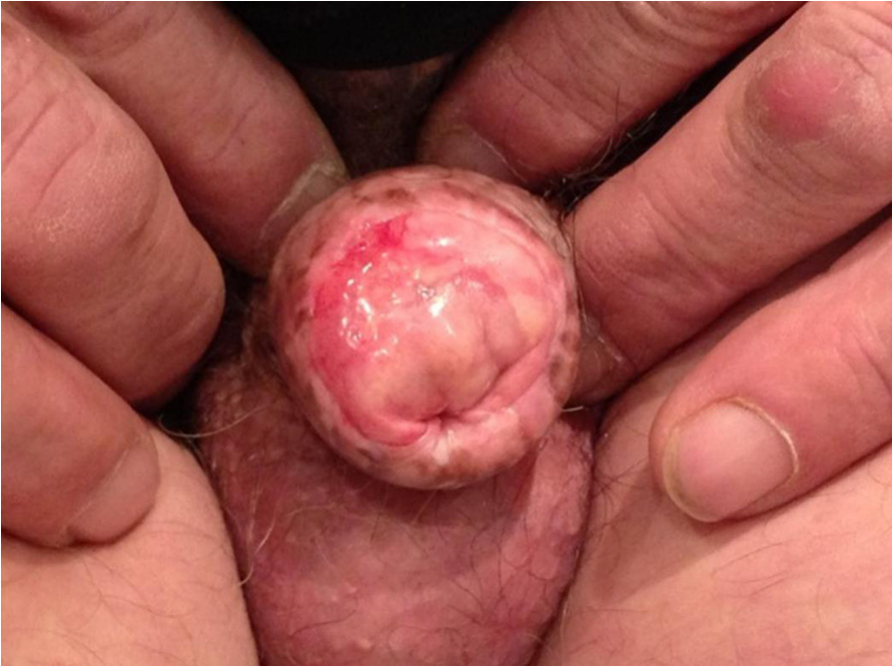
Acute skin toxicity observed 1 month after interstitial high-dose rate brachytherapy.

Evaluation of the urinary functional impact of brachytherapy was performed for all patients by comparing pre and post-operative IPSS scores obtained 1, 6, 12 and 24 months after brachytherapy. While the mean preoperative IPPS score was 4, after a non-significant transient worsening at 1 month post-brachytherapy (IPSS = 6), no significant difference was noted compared to base-line for evaluations at 6 (IPSS = 5), 12 (IPSS = 5) and 24 months (IPSS = 4) (Figure 4A). However, 1 pt (9%) presented an urethral meatus stenosis requiring surgical management by dilatation at 3 and 12 months to obtain a comparable urinary function to preoperative status.Assessment of sexual complications was performed for all patients by comparing pre and post-operative IIEF-5 obtained at 1, 6, 12 and 24 months after brachytherapy. Median pre-operative IIEF-5 was 16, no significant difference was observed at 1 (IIEF5 = 16), 6 (IIEF5 = 13), 12 (IIEF5 = 13) and 24 months (IIEF5 = 13) (Figure 4B). The mean IIEF-5 score at 2 years was 12.2 (SD 7.9) corresponding to moderate erectile dysfunction.Skin appearance observed in the treated area was analyzed comparatively to the pre-treatment status, 1, 6, 12 and 24 months after brachytherapy, by using the CTC v4.0 classification. The assessment of skin toxicities during follow-up showed a significant difference between pre and post-operative assessment at one month (p < 0.01). No other significant differences were found between the different follow-ups at 6 (p = 0.9), 12 (p = 0.7) and 24 months (p = 0.3). Cutaneous complications encountered were mainly grade 1 and 2 radio-dermatitis observed at 1 month in 50% (6 pts) and 41% (5 pts) respectively (Figure 5). One patient (9%) experienced a grade 3 skin necrosis that required 45 sessions of hyperbaric oxygen therapy to obtain complete healing after 17 months while a retractile grade 1 fibrosis of the glans was observed. Grades 1 telangiectasias in or next to the irradiated area were observed in 33% of cases (4 pts).

The penile preservation rate was 91.7% with one patient who presented a local recurrence treated by total amputation of the penile.

## Discussion

In case of localized penile cancer, interstitial brachytherapy after circumcision is considered as the standard conservative treatment [3,4]. Compliance with brachytherapy indication for penile cancer determines the oncological and functional results. In fact, tumor size > 4 cm and stage ≥ T3 (invasion of the urethra) significantly increases the risk of relapse [10-13] and skin necrosis [13,14] after brachytherapy. In addition, the *American Brachytherapy Society* (ABS) and the *Groupe Européen de Curiethérapie of the European Society of Therapeutic Radiation Oncology* (GEC-ESTRO) recommended performing between 3 and 6 procedures per year to be able to offer this treatment modality implying the notion of expert center [11]. Low and pulsed-dose rate (PDR) brachytherapy represented until recent years the reference technique [15,16]. However, the evolution of brachytherapy techniques, after-loading machines, treatment planning software and more stringent rules of radiation safety have positioned high-dose rate brachytherapy as the reference technique for numerous indication such cervical and prostate tumors [5-7,15,17]. But clinical data of HDR brachytherapy for penis cancer remains rare. To our knowledge, we reported clinical and functional results of the largest study of interstitial HDR brachytherapy for localized cancer of the penis.

With a local control rate of 92%, the oncological results reported in this series are comparable to those published in the series of LDR/PDR brachytherapy (Table 2). In these series, the rates of specific survival and local control vary from 72 to 92% and from 77 to 87% respectively [12-20]. In a retrospective study, De Crevoisier et al. [14] presented the results on 144 patients treated with LDR brachytherapy (median total dose of 65 Gy) over a period of 31 years. Local recurrence-free, overall and specific survivals were respectively 80%, 65% and 92%. Rozan et al. [16] reported overall survival rates of 66% and 52%, specific-survival rates of 88% and 88% and relapse-free survival rates of 78% and 67% at 5 and 10 years respectively.

**Table 2:** Comparative clinical outcome analysis from brachytherapy series

**Authors**	**Type**	**n**	**Dose (Gy)**	**F/up (months)**	**LC 5y (%)**	**CSS 5y (%)**	**Necrosis (%)**	**Stenosis (%)**	**PP (%)**
Chaudhary et al. [16]	LDR	23	50	21	70		0	9	70
De Crevoisier et al. [12]	LDR	144	65	68	80(10)	92(10)	26	29	72
Crook et al. [17]	LDR/PDR	67	60	48	87	83,6(10)	12	9	88
Delannes et al. [18]	LDR	51	50-65	65	86	85	23	45	75
Kiltie et al. [19]	LDR	31	63.5	61.5	81	85,5	8	44	75
Mazeron et al. [20]	LDR	50	60-70	36-96	78		6	19	74
Rozan et al. [15]	LDR	184	63	139	86	88	21	45	78
Soria et al. [14]	LDR	102	61-70	111	77	72	1	1	72
Petera J et al. [11]	HDR	10	54*	20	100 (<2y)	100 (<2y)	0	0	100
Present serie	HDR	12	36/39**	27	83	100	9	9	92

Type: modality of radiation therapy; LDR: Low-dose rate brachytherapy; PDR: Pulse-dose rate brachytherapy; HDR: High-dose rate brachytherapy; n: number of patients; LC: local control; CSS: cause specific survival; F/up: follow-up in months; PP: Penile preservation.

*54 Gy in 18 fractions over 9 days.

**36 Gy in 9 fractions over 5 days (in the adjuvant setting: 6 Gy day 1 + 2 x 3.75 Gy from day 2 to day 5) or 39 Gy in 9 fractions over 5 days (in sole therapy: 7 Gy day 1 + 2 x 4 Gy from day 2 to day 5).

The median time to onset of loco-regional recurrence in our study was 7.7 months [6–9.4]. Similar data are reported in the literature with the notion that most of loco-regional recurrences occur in the first two years after treatment [18]. However, De Crevoisier et al. [14] observed 10-year local, inguinal and metastatic recurrence rates of 20%, 11% and 6% respectively. Moreover, Solsona et al. [19] reported a late recurrence rate of 2.5%. It is therefore necessary to recommend a prolonged surveillance and provide education to patients to detect these late local, lymph node and metastatic relapses.

The only series of HDR brachytherapy for penile cancers has been published by Petera et al. [10]. The authors reported the results of a cohort of 10 patients with squamous cell carcinoma of the penile. Eight pts were classified as T1N0M0, 1 pt TisN0M0 and 1 pt T1N1M0. Ninety percent of the tumors were localized to the glans. With a median follow up of 20 months [3.4 - 90.6], the authors reported a loco-regional relapse-free survival rate of 100%. The total delivered dose was 54 Gy (3 Gy/f, 2 fractions/day over 9 days). No patient presented urethral stenosis or penile necrosis. Concerning sexual evaluation, patients retained equivalent results to what was observed preoperatively. Our shorter regimen (5 days) reports similar results in terms of local control while significantly reducing the length of hospitalization.

In case of local relapse after primary brachytherapy, a salvage surgery by partial amputation of the penile, with sufficient safety margin, leads to survival rates without second local recurrence, equivalent to those obtained after surgery for primary tumors. Indeed, salvage surgical series report 10-year specific survival rate ranged from 84% to 92% similar to those described in the case of initial surgery [19,20]. Second relapse rates after salvage radical non-conservative surgery for local relapse range between 0 and 7.1% [19], while the loss of the organ has a deleterious psychological impact.

Regarding functional urinary outcomes, the urethra is considered as the main organ at risk. The issue of brachytherapy is to limit the impact on urinary function while preserving the oncological outcome. The measurement of IPSS at different follow-up visits confirmed the absence of significant degradation of urinary function after HDR brachytherapy. In the literature, various studies of LDR/PDR brachytherapy evaluated post-brachytherapy urinary status by the presence or absence of urethral stenosis (most serious urinary complication). In our series, the rate of urinary stenosis was 9% (1 pt), whereas in the series of LDR/PDR brachytherapy, this rate varies between 8 and 45% [12-14,16,21-23]. Generally, stenosis is treated by dilatation or endoscopically. While urethral stenosis remains the major side effect after brachytherapy, the possibilities of optimizing the dose distribution to the urethra offered by PDR/HDR brachytherapy can reduce the risk of urinary side effects.

In our study, post-brachytherapy IIEF-5 score evaluation compared to the pre-therapeutic status confirmed the absence of significant deterioration of the sexual function after HDR brachytherapy. Although penile cancer and its treatment can have a negative impact on sexual function, this functional aspect is poorly documented in the literature. Maddineni et al. [24] reported that mutilating treatment for penile cancer caused a significant decline in well-being and an increase of pathological anxiety for more than 30% of patients and psychiatric symptoms for 50% of patients. Two thirds of patients reported a decrease in sexual activity after mutilating surgery. In a retrospective study, Crook et al. [25] reported a conservation rate of sexual function of 40%. In their series of HDR brachytherapy, Petera et al. [10] reported that 90% of patients preserved their sexual function at the end of follow-up. Interestingly, in a surgical study of 18 patients treated by partial amputation, Romero et al. [26] reported that 33% of patients conserved a similar sexual status compared to the preoperative period.

After penile brachytherapy, the most severe late skin complication is necrosis. In our series, 1 pt (9%) who received the highest dose (43 Gy; EQD2αβ3 68 Gy), presented skin necrosis successfully treated with hyperbaric oxygen. Gomez-Iturriaga et al. [27] used also successfully hyperbaric oxygen therapy in 7 pts with skin necrosis post-brachytherapy. In the literature, the rate of skin necrosis ranged between 0 to 26% of cases depending on the series [12-14,16,21-23] while this complication has never been described after surgery. However, acute skin complications are much more common. In our series, 90% of patients had radio dermatitis (G2 41%). This is a classic acute complication after radiation therapy for which the patient must be informed. This complication should heal within 3 months after brachytherapy. Other lesions observed in the treated area were hyper-pigmentation and telangiectasia (common complications after skin irradiation). Crook et al. [25] described low-grade cutaneous complications such as hypo-pigmentation, telangiectasia and fibrosis in 33% of cases. De Crevoisier et al. [14] reported a 10-year painful ulceration rate of 26%. In the immediate post-brachytherapy period, Petera et al. [10] noticed that radio dermatitis resolved in 8 weeks, while the authors did not observe any skin necrosis. This last result could be explained by the fact that the median CTV in the Petera study was 7 cc versus 12.1 cc in our series.

In our study, 1 pt (9%) required total amputation of the penis due to local relapse leading to an organ preservation rate of 92%. In the literature, this rate ranged between 72 and 88% [12-14,16,21-23].

The limitations of our study are essentially represented by a small number of patients (12) and a short median follow-up (27 months), which is not sufficient to precisely analyze the rate of late relapses occurring after the fifth year of follow-up. On the other hand, objective analysis of post-brachytherapy urinary function would have required before and after brachytherapy, the use of uroflowmetry which remains in this field the standard evaluation tool.

## Conclusion

For localized cancers penile (T1-2), brachytherapy after circumcision represented the treatment of choice. Because of its ability to optimize the dose distribution and its low constraints in terms of radiation protection, HDR brachytherapy gradually gains in popularity. For the conservative treatment of penile cancer, this technique seems to give promising oncological and functional results even if the follow-up is too short to allow an accurate comparison to the results published for LDR/PDR brachytherapy. These results need to be confirmed by the publication of larger series of patients with extended follow-up.

### Consent

Written informed consent was obtained from the patient for the publication of this report and any accompanying images.

## Competing interests

The authors declare that they have no competing interests.

## Authors’ contributions

YR analyzed the clinical data and wrote the manuscript; AF revised all the manuscript; MD revised the “Functional results” section; JG managed the statistical analysis; MEC revised the “Dosimetric results” and “HDR brachytherapy delivery” sections; MG revised the “Dosimetric results” and “Planification” sections; AM revised the “Functional results” section; DC revised the “Discussion” section; JA revised the “Discussion” section; JMHL conceived, planned the study and wrote the manuscript. All authors read and approved the final manuscript.
